# Efficacy and cost-effectiveness of nutritional intervention in elderly after hip fracture: design of a randomized controlled trial

**DOI:** 10.1186/1471-2458-10-212

**Published:** 2010-04-27

**Authors:** Caroline E Wyers, José JL Breedveld-Peters, Petronella LM Reijven, Svenjhalmar van Helden, Nick A Guldemond, Johan L Severens, Aart D Verburg, Berry Meesters, Lodewijk W van Rhijn, Pieter C Dagnelie

**Affiliations:** 1 Department of Epidemiology, Maastricht University Medical Centre, PO Box 616, 6200 MD Maastricht, The Netherlands; 2 Department of Dietetics, Maastricht University Medical Centre, PO Box 5800, 6202 AZ Maastricht, The Netherlands; 3 Department of Trauma surgery, Maastricht University Medical Centre, PO Box 5800, 6202 AZ Maastricht, The Netherlands; 4 Department of Orthopaedic Surgery, Maastricht University Medical Centre, PO Box 5800, 6202 AZ Maastricht, The Netherlands; 5 Department of Health Organization, Policy and Economics, Maastricht University Medical Centre, and Institute of Health Policy and Management, Erasmus University Rotterdam, PO Box 616, 6200 MD Maastricht, The Netherlands; 6 Department of Orthopaedic Surgery, Orbis Medical Centre, PO Box 5500, 6130 MB Sittard, The Netherlands; 7 Department of Surgery, Atrium Medical Centre, PO Box 4446, 6401 CX Heerlen, The Netherlands

## Abstract

**Background:**

Hip fracture patients often have an impaired nutritional status at the time of fracture, which can result in a higher complication rate, prolonged rehabilitation time and increased mortality. A study was designed to evaluate the effect of nutritional intervention on nutritional status, functional status, total length of stay, postoperative complications and cost-effectiveness.

**Methods:**

Open-labelled, multi-centre, randomized controlled trial in hip fracture patients aged 55 years and above. The intervention group receives dietetic counselling (by regular home visits and telephone calls) and oral nutritional supplementation for three months after surgery. The control group receives usual dietetic care as provided by the hospital. Outcome assessment is performed at three and six months after hip fracture.

**Discussion:**

Patient recruitment has started in July 2007 and has ended in December 2009. First results are expected in 2011.

**Trial registration:**

ClinicalTrials.gov NCT00523575

## Background

In the elderly, the incidence of hip fractures is high and it will increase in the nearby future due to the changes in the age demographics, the increased life expectancy and the continuous improvement of health care [1,2]. Hip fractures are one of the most common reasons for hospital admission and transfers to nursing home [3]. Only 37% of the hip fracture patients will return to their pre-fracture functional status, leading to high health care costs and a major burden on health care utilization. These costs are not only determined by acute hospital costs, but even more by the long term costs such as recovery in rehabilitation clinics, the need for home care and the increased burden on informal care givers [4,5].

At the time of hospitalization for a hip fracture, the prevalence of malnutrition ranges from 2% [6] to 63% [7]. Poor nutritional status in hip fracture patients is associated with impaired muscle function, disability [8], loss of independency, lower mental function, decreased quality of life [8], delayed wound healing, higher complication rate [9,10], prolonged rehabilitation time [8,9,11] and increased mortality both during and after hospital admission [9,12-16]. During hospital admission, the nutritional status can deteriorate further due to increased energy expenditure caused by metabolic stress, combined with a low intake due to the lack of appetite, nausea and psychological factors.

In the past decades, several studies have been conducted to determine the effectiveness of various types of nutritional intervention in elderly hip fracture patients on the length of stay, mortality, complications, nutritional and functional status. Results of these studies are inconsistent and the evidence for nutritional supplementation remains limited [17]. Oral nutritional supplementation is the simplest type of nutritional intervention for hip fracture patients to improve the energy and protein intake and nutritional status, although compliance is often poor [18]. Personal attention from a dietetic assistant can improve compliance with and tolerance of nutritional supplements [19] and help to establish a prolonged effect of the nutritional intervention.

The aim of the present study is to investigate the effect of intensive dietary intervention, comprising a combination of dietetic counselling and oral nutritional supplementation during hospitalization and after discharge, on the nutritional status, total length of stay and health care costs after hip fracture. We hypothesize that the combination of dietetic counselling and oral nutritional supplementation in hip fracture patients will improve patients' energy and protein intake, improve their nutritional status, reduce the number of complications and total length of stay in hospital and rehabilitation clinics, and lower health care costs.

## Methods

### Study design, general outline and randomization

**Figure 1:**
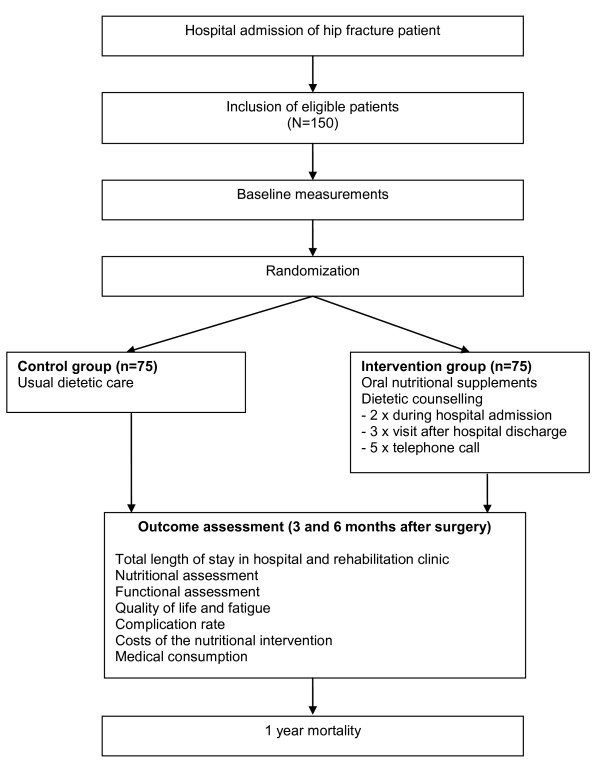
**Study design**.

Figure 1 shows the design of the study, which is an open-label, randomized controlled, multi-centre trial. Patient allocation to intervention or control group is performed after stratification for hospital, gender, and age (55-74 years vs. 75 years and above). Patients allocated to the intervention group receive dietetic counselling and oral nutritional supplementation for 3 months following surgery. The control group receives usual nutritional care. Patients are enrolled within 5 days after surgical treatment of hip fracture, and baseline measurements are performed immediately after enrolment. Outcome measurements are performed at the patient's home at three and six months following hip fracture. The study has been approved by the Medical Ethical Committee of Maastricht University Medical Centre and Maastricht University and is conducted according to the Declaration of Helsinki.

### Study population and recruitment of the study population

For patient recruitment, a daily inventory is made of hip fracture patients admitted to the surgical and orthopaedic wards of three hospitals in South-Limburg in The Netherlands: Maastricht University Medical Centre, Maastricht (MUMC); Atrium Medical Centre, Heerlen (AMC); and Orbis Medical Centre, Sittard (OMC). Based on this inventory, eligible candidates are invited to participate and written informed consent is obtained within 5 days after surgery. Inclusion criteria are aged 55 years and above and hospital admission for surgical treatment of hip fracture. Patients are excluded if they have a pathological or periprosthetic fracture; disease of the bone metabolism (e.g. M Paget, M Kahler, hyperparathyroidism); life expectancy of less than 1 year due to underlying disease (e.g. cancer); use oral nutritional supplements before hospital admission; are unable to speak Dutch; live outside the region of South-Limburg, or are bedridden before the hip fracture. Patients who suffer from dementia or who are cognitively impaired according to the Abbreviated Mental Test (AMT) (a score of less than 7) are also excluded [20,21].

### Nutritional intervention

The nutritional intervention is a combination of dietetic counselling and oral nutritional supplements for three months. The intervention starts during hospital admission and continues after discharge during the stay at the rehabilitation clinic or at the patient's home. During hospitalization, the study dietician visits the patient twice. At the first visit, two to five days after surgery and immediately after baseline measurements, the dietician interviews the patient regarding medical and social status, and pre-fracture mobility. The dietician also performs a 24-hour recall and takes a general dietary history of the patients' diet before hospitalization. Next, the patient receives the nutritional supplement, a milk-based supplement providing 21 kJ (500 kcal) and 40 g of protein. The dietician advices the patient on the consumption of the supplement and arranges extra care or services to optimize the food intake if necessary. Before hospital discharge, the dietician visits the patient for the second time. During this visit, a 24-hour recall is performed and the consumption of the nutritional supplement is evaluated. Furthermore, arrangements are made to continue the dietetic advice and the consumption of the nutritional supplement at home or during the stay at the rehabilitation clinic. At home or during the stay in a rehabilitation clinic, the dietician visits the patient three times (one week, two weeks and six weeks after discharge) and makes five telephone calls with the patient (three, four, five, eight and ten weeks after discharge). During these visits, food intake and supplement use is assessed by a 24-hour dietary recall, and tailor-made dietetic advice is given to optimize the amount and composition of the diet. As soon as the patient meets nutritional requirements with a normal diet, the use of the nutritional supplement is stopped. Compliance with the nutritional supplement is evaluated by the 24-hour dietary recalls, patients' registration of the consumption in a diary and by collecting the capsules of the cans of the nutritional supplement during the home visits.

### Usual care

Patients allocated to the control group receive usual care as provided in the hospital, rehabilitation clinic or at home, i.e. dietetic care or nutritional supplements are only provided on demand of the medical doctor in charge.

### Outcome assessment

The primary outcome measure is total length of stay in hospital and rehabilitation clinics including hospital readmissions. Secondary outcome measures, assessed at three and six months after hip fracture, are nutritional status, functional status, quality of life, complication rate and one-year mortality.

Assessment of nutritional status includes total food and supplement intake, measurement of body composition, muscle strength and biochemical parameters in blood. Anthropometric measurements are body weight, height, thickness of biceps, triceps, sub scapula and supra-iliac skin fold, and circumference of mid-arm, waist and hip. Body composition is measured by bio-electrical impedance spectroscopy. Biochemical parameters include albumin, pre-albumin, CRP, haemoglobin and hematocrit in blood.

Functional status is measured by the Groningen Activity Restriction Scale (GARS) [22], which assesses disability with regard to (instrumental) activities of daily living, and by the Harris Hip Score to evaluate changes in hip function.

Quality of life is measured using the EuroQoL [23,24] and the Medical Outcomes Study 36-item Short-Form Health Survey [25,26]. Mental state and depression is assessed by the Mini-Mental State Examination (MMSE) and the Hospital Anxiety and Depression Scale (HADS) [27,28]. To measure fatigue, the Checklist Individual Strength (CIS) [29] is used.

### Confounders

Pre-fracture information on co-morbidity, medication use and fracture history is obtained by interviewing the patient. Information on type of hip fracture, surgical treatment, admission and discharge dates, and post-operative complications are obtained from medical charts.

### Economic evaluation

The costs analysis will compare the costs of the nutritional intervention and the usual care over a 6-month period [30]. Medical and non-medical costs are obtained from a 3-month retrospective standardized costing questionnaire. Health care costs will be estimated according to the Dutch guideline for cots-analysis in health care research [31]. Incremental costs between the strategies will be related to a difference in outcome during 6 months follow up and being expressed in a incremental cost-effectiveness ratio. Statistical uncertainty will be assessed using bootstrap simulations [32].

### Process evaluation

A process evaluation is carried out to evaluate whether the nutritional intervention follows the protocol, with regard to the dietetic counselling and the consumption of the nutritional supplement. The feasibility of the nutritional intervention program is evaluated through summative evaluation with the identified stakeholders being patients and health care providers (medical doctors, dieticians, nurses). Experiences and opinions on feasibility, barriers and stimulating or facilitating factors for implementing the nutritional intervention in the transmural care are identified from the stakeholders' perspective. Data are collected by structured interviews with patients and in-depth interviews in a representation of patients and health care providers. A subsample of health care providers is asked to participate in a focus group meeting. The results of the process evaluation will be used as a basis for successful implementation of the nutritional intervention program in the transmural care for hip fracture patients.

### Sample size/power calculation

Nutritional intervention in hip fracture patients can result in an estimated reduction in total length of hospital and rehabilitation stay by 31.3% (SD: 59.0%). A sample of 75 patients per treatment arm is sufficient to detect this effect with a power of 90% and a two tailed alpha of 0.05. To detect a between-group difference in weight of 2.13 kg (SD: 3.72 kg), a number of 61 patients per treatment arm is sufficient using the same power and alpha.

### Statistical analysis

The intervention effects of primary and secondary outcome measures will be analyzed according to the intention-to-treat principle. In addition, a secondary per protocol analysis will be performed in patients with adequate compliance. After initial univariate analyses of differences in the primary outcome between the intervention and control group, multivariate analyses will be used to adjust for potential confounders such as age, gender, baseline values of nutritional status, physical disability, co-morbidity, and mental state including depression.

## Discussion

Patient recruitment has started in July 2007 and has ended in December 2009. Follow-up of the patients is to be completed in June 2010, except for data of one-year mortality. First results are expected in 2011. We expect that this study will answer the question whether nutritional intervention in hip fracture patients improves their nutritional and functional status, resulting in a shorter length of stay in hospital and rehabilitation clinics, fewer postoperative complications, lower mortality, and cost-effectiveness.

## Competing interests

The authors declare that they have no competing interests.

## Authors' contributions

PR, SH and PD have designed the study. CW participated in patient recruitment, data acquisition and has written the draft of this manuscript. JB participated in data acquisition and conducted the process evaluation. SH, NG, LR, AV and BM were responsible for patient recruitment. JS supervised the economic evaluation.

All authors have read and approved the final version of the manuscript.

## Pre-publication history

The pre-publication history for this paper can be accessed here:

http://www.biomedcentral.com/1471-2458/10/212/prepub
